# Gut microbiota as key mediators of animal acclimation to temperature changes: mechanisms and interventions

**DOI:** 10.1128/aem.02412-25

**Published:** 2026-07-06

**Authors:** Shuyu Zhang, Jianing Tu, Weichen Hong, Jiaxin Liu, Chenyu Xue, Na Dong

**Affiliations:** 1 Laboratory of Molecular Nutrition and lmmunity, College of Animal Science and Technology, Northeast Agricultural University 12430, Harbin, People's Republic of China; The Pennsylvania State University, University Park, Pennsylvania, USA

**Keywords:** gut microbiota, temperature change, microbiota-gut-brain axis, mitigation strategies

## Abstract

With the intensification of global climate change, temperature fluctuations profoundly affect animal physiology and health. Research has shown that the gut microbiota, as a critical bridge between the host and its environment, helps animals adapt to temperature changes by regulating intestinal barrier stability, immune function, and energy metabolism. This adaptive capacity underscores the indispensable role of gut microbiota in temperature change responses. In cold environments, animals increase food intake and activate brown adipose tissue to maintain body temperature, but prolonged exposure causes metabolic overload and gut microbiota imbalance. Chronic cold reduces beneficial bacteria and increases pro-inflammatory species, impairing intestinal barrier integrity and inducing systemic inflammation, ultimately leading to metabolic disorders and immunosuppression. Similarly, heat exposure leads to pathogenic overgrowth and immune dysfunction, reducing microbial diversity and increasing the abundance of harmful bacteria, ultimately impairing animal health. Furthermore, the gut-brain axis plays a central role in coping with environmental stress, as temperature change alters microbial composition and metabolites, impacting neurotransmitter synthesis and release, thereby regulating physiological states and emotional responses. Finally, targeted microbial interventions—such as fecal microbiota transplantation (FMT), probiotics, prebiotics, synbiotics, and postbiotics—are discussed as effective strategies to restore gut microbiota homeostasis, enhance host resilience to temperature change, and improve animal health under temperature fluctuations.

## INTRODUCTION

Environmental temperature fluctuations, a core feature of climate in general, have been exacerbated by global climate change, and thus pose increasingly severe challenges to animal health and agricultural productivity. Temperature variations not only directly disrupt energy metabolism and physiological homeostasis in animals but also indirectly affect host immune responses, neural regulation, and environmental adaptability by reshaping the structure and function of the gut microbiota (1, 2). For instance, under high-temperature conditions, compromised intestinal barrier integrity can trigger systemic inflammation and reduce key indicators of animal production (e.g., growth rate, feed conversion efficiency, and product quality), while low-temperature exposure alters energy allocation efficiency through microbial metabolic reprogramming, increasing metabolic costs for thermoregulation (3, 4). Understanding such temperature-microbiota-host interactions is therefore highly meaningful for optimizing livestock breeding strategies and improving agricultural sustainability, and further in-depth investigation is warranted to develop effective countermeasures against the challenges brought by climate change.

Recent studies reveal that temperature change exerts regulatory effects on the metabolic activities of the gut microbiota, with divergent impacts on key processes, including short-chain fatty acid synthesis, bile acid transformation, and amino acid metabolism. Low temperatures promote the proliferation of *Firmicutes* and enhance secondary bile acid production, activating thermogenic programs in brown adipose tissue (BAT) (5, 6). Conversely, high temperatures suppress intestinal tryptophan metabolism, reducing the biosynthesis of neurotransmitter precursors such as serotonin (5-HT), thereby influencing stress-related behaviors via the gut-brain axis (7, 8). Notably, microbial metabolites—including short-chain fatty acids (SCFAs) and indole derivatives—bidirectionally regulate host energy metabolism and immune homeostasis through G protein-coupled receptors (GPCRs) and nuclear receptor signaling (9). Building on these mechanistic insights, current targeted microbial interventions, such as specific probiotic strains, synbiotics, and postbiotics, have demonstrated preliminary potential in mitigating temperature change-induced intestinal damage. For example, FMT restores butyrate-producing bacterial abundance to alleviate heat stress-induced intestinal leakage in mice (10), while *Lactobacillus plantarum* supplementation enhances cold acclimation in weaning piglets by modulating bile acid metabolism (11). These advances provide novel perspectives for improving animal environmental resilience through precision microbial modulation.

This review explores how the gut microbial community undergoes compositional restructuring and coordinates the functional responses (e.g., inflammatory modulation, barrier protection, and metabolic acclimation) of diverse microbial taxa under temperature shifts, focusing on the key role of microbial metabolites in gut-brain axis communication, energy balance, and immune regulation. By analyzing advanced intervention studies, we assess the potential of microbiota-targeted approaches to reduce temperature stress, providing scientific insights for improving animal health management amid temperature fluctuations.

## GUT MICROBIOTA RESPONSE TO TEMPERATURE CHANGES

The stability and function of the gut microbiota are important for maintaining host health. Temperature changes, as a critical environmental factor, can significantly affect the composition and metabolic activities of gut microbiota. Specifically, temperature fluctuations not only lead to the restructuring of microbial communities by altering the relative abundance and diversity of different taxa but also influence the levels of key metabolites, such as SCFAs and bile acids, through modulation of microbial metabolic pathways.

### Restructuring of gut microbiota composition

    Temperature changes significantly alter the composition, diversity, and metabolic activities of the gut microbial community, serving as a foundational link between environmental signals and host acclimation (12, 13). Notably, this interaction is not unidirectional but co-regulated by ambient temperature and intrinsic host traits (14). It exhibits common response rules across species and significant specificity due to differences in host taxa and genotypes, forming a tripartite interaction network among the environment, host, and gut microbiota, which provides a core basis for subsequent metabolic regulation and temperature acclimation phenotypes.

#### Differential gut microbiota responses to temperature stress between endotherms and ectotherms

Temperature fluctuations drive changes in the taxonomic abundance of the gut microbial community in both endotherms and ectotherms, and their response patterns differ (12, 15). Notably, these shifts in microbial α-diversity and taxonomic abundance merely reflect structural changes in the gut microbial community, and a single diversity index cannot reliably indicate the health status of the microbiome or host physiological homeostasis (16). In endotherms (e.g., mice, pigs, and livestock), short-term cold exposure may increase microbial α-diversity, while prolonged exposure reduces overall community richness (13, 17–20). It generally promotes the proliferation of *Firmicutes* and inhibits *Bacteroidetes*, along with increased abundance of opportunistic pathogens such as *Helicobacter pylori* and decreased butyrate-producing bacteria like *Lachnospiraceae* (21–24). Heat exposure significantly reduces the abundance of beneficial taxa such as *Lactobacillus* and increases the proportion of pathogens including *Proteobacteria* and *Salmonella*, disrupting gut homeostasis (25–27).

In ectotherms (e.g., fish, reptiles, and insects), the gut microbial response to temperature stress is more intense (distinct from endotherms). Under cold stress, α-diversity generally decreases significantly (without the “short-term increase” trend observed in endotherms), and changes in dominant phyla are species-specific (28). After cold exposure in freshwater tilapia (*Oreochromis niloticus*), the abundance of *Actinobacteria* increases and *Firmicutes* decreases significantly, with enzyme-producing bacteria such as starch-degrading bacteria enriched to improve energy utilization efficiency (29). Honeybees (*Apis mellifera*) show a sharp decline in the abundance of beneficial bacteria such as *Lactobacillus*, with an increased colonization risk of pathogenic fungi (e.g., *Beauveria bassiana*) (30). Under heat stress, ectotherms exhibit a much higher degree of gut microbial dysbiosis compared to endotherms, which is primarily manifested by a sharp increase in the relative abundance of *Proteobacteria* rather than a single change in diversity indices. *Proteobacteria* becomes the dominant microbiota in Atlantic salmon (*Salmo salar*) after heat exposure, with pathogens such as Vibrionaceae dominating the community structure (31). When western fence lizards (*Sceloporus occidentalis*) were exposed to high temperatures, the abundance of *Firmicutes* decreased significantly, the abundance of predicted pathogenic clades increased, and the rate of gut microbial compositional turnover accelerated (32).

The core reason for this difference is that ectotherms cannot independently regulate their body temperature, and the intestinal microenvironment (temperature, pH) fluctuates sharply with the external environment, leading to rapid turnover of microbial taxa, while endotherms can buffer changes in the intestinal microenvironment through thermoregulation (33). These contrasting responses thus provide a clear, unified perspective for understanding how host thermoregulatory strategies shape gut microbial stability under temperature stress and help establish a more consistent framework for interpreting temperature-driven microbiota shifts across different animal groups.

#### Intrinsic genotypic differences shape gut microbiota-mediated temperature acclimation

The common microbial response patterns induced by temperature are not entirely dominated by the environment. Host genotype, a key intrinsic factor, significantly regulates the intensity, direction, and functional correlation of microbial responses, thereby leading to divergent temperature acclimation phenotypes among different individuals or species. This regulatory effect is prevalent in both endotherms and ectotherms (34–36). Cold-tolerant Hulunbuir sheep and cold-sensitive Hu sheep, both endotherms, exhibit inherent gut microbial differences due to genotypic divergence. *Prevotella* (propionate-producing) and *Roseburia* (butyrate-producing) are enriched in Hulunbuir sheep, enabling “energy-efficient cold acclimation” via hepatic gluconeogenesis and intestinal barrier reinforcement under low temperatures. In contrast, Hu sheep have a higher proportion of acetate-producing microbiota and rely on acetate-driven thermogenesis with greater energy loss and reduced growth (37). Among ectotherms, genotypic differences between temperate honeybees (*Apis cerana* and *A. mellifera*) and tropical honeybees also shape distinct microbial traits. Temperate honeybees harbor enriched gut symbiont Gilliamella, which optimizes energy metabolism to enhance cold acclimation and reduce microbial dysbiosis under heat stress, while tropical honeybees lacking Gilliamella show significantly weaker tolerance to temperature fluctuations (34).

These observations illustrate how host genotype shapes the composition of the gut microbiota and its contribution to thermal acclimation. This knowledge not only helps explain differences in temperature tolerance among individuals and species, but also offers practical clues for breeding programs and targeted microbial interventions aimed at improving thermal resilience in farmed and managed animals.

### Microbial metabolite network reconfiguration

Notably, temperature-associated alterations in gut microbial metabolism have been documented in both endotherms and ectotherms, including fish and reptiles. However, in-depth studies exploring the precise molecular mechanisms and functional consequences underlying microbial metabolic regulation during temperature stress are predominantly concentrated in endotherms, including agriculturally important livestock, such as pigs, chickens, and cattle, as well as classical mammalian models such as mice. Due to their key roles in livestock production, translational relevance for biomedical research, and unique thermoregulatory strategy of maintaining stable core body temperature through metabolic heat production, endotherms serve as important and well-characterized models for understanding microbe-mediated adaptive responses to temperature stress. Therefore, this section focuses on the microbial metabolic responses and underlying regulatory pathways established in endotherms (38).

Temperature stress not only reshapes gut microbial community structure but also triggers marked changes in microbial metabolic activity in endotherms (26). These metabolic changes are mediated by shifts in the production of key microbial metabolites, such as SCFAs, bile acids, and amino acids. These metabolites mediate the interaction between gut microbiota and the host. They play crucial roles in regulating host energy metabolism, immune function, and gut homeostasis (39–41). For example, SCFAs contribute to environmental adaptation by modulating energy balance; bile acids are central to lipid metabolism; and amino acids and their derivatives participate in neurotransmitter synthesis and energy supply. Within endothermic hosts, dynamic changes in these metabolites directly reflect the host’s adaptive capacity to temperature change and further affect host energy metabolism and gut health (42, 43).

#### SCFAs: key regulators of energy and immunity

SCFAs are important for regulating energy metabolism, maintaining gut homeostasis, and exerting anti-inflammatory effects (44). The effect of temperature on SCFA production may vary depending on experimental conditions, animal species, and exposure duration. The observed fluctuations in SCFA secretion are attributable to shifts in gut microbial communities. For example, cold exposure increases the abundance of certain bacteria, such as members of the *Clostridiales* order (a family within *Firmicutes*), which are capable of enhancing SCFA production, thus helping to regulate host energy metabolism and body temperature (19, 22). The increased SCFA production is also believed to contribute to the animals' increased food intake to obtain more energy for thermoregulation (45, 46). In one study, exposing mice to 7°C for 3 h during their active phase suppressed the production of SCFAs, including acetate and propionate. This led to an increase in gut pH, disrupting the mildly acidic environment in the gut, thus promoting the growth of harmful bacteria (36).

Heat exposure has been shown to reduce SCFAs levels in some animals. For example, heat-exposed sows during late gestation exhibited a significant reduction in fecal propionate, butyrate, and total SCFAs, while acetate levels remained unchanged (47). In heat-exposed broilers, the cecal content showed an increase in acetate levels, which may be associated with the increased proportion of *Ruminococcaceae* and *Clostridia* (48). The reduction in SCFAs is primarily associated with host energy metabolism imbalance and increased oxidative stress. These findings demonstrate that gut microbiota dynamically modulate SCFA production in response to temperature changes in endotherms, with distinct patterns under cold versus heat exposure. These temperature-induced shifts in SCFA profiles not only reflect microbial acclimation to temperature stress (with long-term health consequences yet to be explored) but also serve as predictable indicators of the gut microbiota’s adaptive state. Given their direct influence on energy supply and inflammatory status, monitoring SCFAs can help assess how effectively animals cope with temperature challenges, and manipulating the gut microbiota to sustain stable SCFA levels may offer a practical approach to enhancing thermal tolerance in livestock and other managed animals.

#### Bile acids: linking metabolism and immune modulation

Bile acids play critical roles in lipid digestion and absorption, cholesterol metabolism, and maintaining gut health. Their secretion and metabolism are also influenced by environmental temperature changes in endotherms. Primary bile acids are synthesized primarily in the liver from cholesterol through a series of enzymatic reactions. Secondary bile acids are converted from primary bile acids by specific gut microorganisms including *Clostridium*, *Bacteroides*, *Roseburia*, and *Faecalibacterium* via intestinal microbial transformation and exert more prominent effects on immune regulation and energy metabolism (49). The influence of temperature appears more pronounced on secondary than primary bile acids, owing to the temperature-dependent nature of gut microbial metabolism responsible for the bioconversion of primary to secondary bile acids. Heat exposure can lead to dysbiosis, which impacts the generation and metabolism of secondary bile acids. For example, after heat exposure, the relative abundance of *Mucispirillum* in bulls decreased, which led to a reduction in secondary bile acids and impacted vitamin A absorption, thus decreasing sperm quality (50). Chronic cold exposure reshapes the gut microbial community in mice and pigs, suppressing the abundance of *Firmicutes* and *Bacteroides* that mediate the biotransformation of primary bile acids, thereby reducing the production of secondary bile acids (6). Meanwhile, cold exposure activates the hepatic CYP7B1-mediated alternative bile acid synthesis pathway, accelerating cholesterol conversion into bile acids and promoting thermogenesis in brown adipose tissue to support adaptive energy metabolism under low-temperature conditions (46).

In summary, temperature stress disrupts bile acid metabolism in endotherms mainly by altering gut microbial function. The resulting changes in bile acid pools further regulate host lipid metabolism and energy homeostasis.

#### Amino acids: key mediators of temperature acclimation and physiological homeostasis

Temperature exposure modulates host amino acid metabolism largely through remodeling the functional capacity of the gut microbiota. In endotherms, temperature-driven shifts in microbial functional guilds are closely associated with changes in amino acid uptake and catabolism, which support adaptive metabolic responses to temperature challenges (51).

Cold exposure enriches microbial taxa specialized in amino acid salvage and nitrogen recycling, a recently recognized mechanism that helps hosts conserve essential amino acids under energy-limited conditions. Prominent examples include urea-degrading bacteria such as Alistipes, as well as members of *Lachnospiraceae* and *Ruminococcaceae*, which collectively drive efficient nitrogen recycling and amino acid production. These microbes enhance the breakdown and utilization of dietary and host-derived nitrogenous compounds, increasing pools of aromatic and branched-chain amino acids to support thermogenesis and systemic energy supply (18, 52–54). Cold-adapted microbiota also modulate tryptophan-kynurenine metabolic flux, with certain *Clostridia* taxa shown to promote neurotransmitter production and redox balance, thereby reinforcing metabolic and neurological acclimation to low temperatures (21, 55, 56).

Heat exposure induces a functional shift in the gut microbiota characterized by suppressed amino acid anabolism and enhanced stress tolerance (57, 58). Specifically, the reduced abundance of beneficial bacteria such as *Lactobacillus* and *Faecalibacterium* downregulates microbial pathways for amino acid synthesis and transport while promoting amino acid deamination and glutamate metabolism. Together with impaired intestinal peptide transporter activity, these changes decrease host amino acid availability and redirect metabolic resources toward stress resistance rather than anabolic growth (42, 59, 60). Most of these microbially driven metabolic changes are reversible under thermoneutral conditions, supporting a buffering role of the gut microbiota in maintaining host homeostasis during transient thermal stress (42, 61).

In summary, the gut microbiota critically regulates host amino acid metabolism under both cold and heat stress. By reshaping nitrogen utilization, amino acid synthesis, and metabolic flux, the microbiota supports energy adaptation and physiological homeostasis, serving as a core mediator of host acclimation to temperature fluctuations (Table 1).

**TABLE 1:** Comparative effects of cold and heat exposure on gut microbiota composition and metabolic pathways

Impact dimension	Cold exposure	Heat exposure
α-diversity	Short-term exposure may increase α-diversity; long-term exposure significantly decreases α-diversity	Short-term exposure transiently increases α-diversity; long-term exposure significantly decreases α-diversity
β-diversity	Long-term exposure significantly alters microbial structure	Long-term exposure significantly alters microbial structure
Dominant phylum changes	*Firmicutes*↑; *Bacteroidetes*↓; *Verrucomicrobia*↓	*Proteobacteria*↑;* Bacteroidetes*↓; *Actinobacteria*↑
Key functional genera	*Lachnospiraceae*↓*; Helicobacter*↑	*Lactobacillus*↓; *Vibrio* and *Photobacterium*↑*; Turicibacter*↑
SCFA changes	Acetate↑ (partial animals); propionate↓ (intermittent exposure in mice); butyrate↓ (long-term exposure)	Total SCFAs↓ (pregnant sows); butyrate↓ (rat HT group); acetate↑ (poultry)
Bile acid metabolism	Secondary bile acids (THDCA)↓; altered hepatic bile acid synthesis pathways	Secondary bile acids↓ (reduced *Mucispirillum* in cattle); impaired vitamin A absorption
Amino acid metabolism	Lysine degradation products (α-AA)↑ (mouse FT group); altered dopamine metabolites	Aspartate (umami)↓; phenylalanine (bitterness)↑ (fish muscle metabolome)

## GUT MICROBIOTA-HOST CROSSTALK IN TEMPERATURE STRESS RESPONSES

Temperature change profoundly impacts host physiology through complex interactions with gut microbiota and their metabolites. Under cold or heat exposure, gut microbiota modulate host acclimation by regulating energy metabolism, immune responses, and thermoregulation. For example, cold exposure triggers host thermogenic programs, which are further enhanced by gut microbiota and their metabolites that promote the activation of BAT and the browning of white adipose tissue (WAT). These processes are supported by microbial activities that influence host energy utilization and heat production. Notably, gut microbiota also initiate long-distance signaling via the gut-brain axis, allowing environmental temperature cues to shape brain activity and behavior (62).

### Microbial-mediated gut-brain axis effects

The gut-brain axis acts as a bidirectional communication network that translates microbial signals into coordinated host adaptive responses under temperature stress. Key regulatory modules include the hypothalamic-pituitary-adrenal (HPA) axis for stress hormone release, the vagus nerve for signal transmission, neurotransmitter balance for neural regulation, and the thyroid hormone axis for metabolic and thermogenic control. Together, these interconnected pathways enable the host to effectively cope with temperature fluctuations and maintain internal homeostasis.

#### HPA axis and stress responses

The gut-brain axis mediates dynamic interactions between the brain and the gastrointestinal tract through bidirectional signaling, encompassing top-down neuroendocrine regulation and bottom-up metabolic and immune feedback (63). The HPA axis integrates temperature signals from cold and heat exposure to drive systemic physiological acclimation (64, 65). Elevated temperatures activate peripheral TRPV1 channels, transmitting signals via the vagus nerve to the hypothalamic preoptic area, while low temperatures engage TRPM8 channels to trigger the sympathetic-adrenal-medullary axis (66, 67). Both pathways converge at the hypothalamic paraventricular nucleus (PVN), stimulating corticotropin-releasing hormone (CRH) secretion, which promotes glucocorticoid (e.g., corticosterone or cortisol) and norepinephrine (NE) release from the pituitary-adrenal axis (68, 69). In hypothermic conditions, NE reduces intestinal blood flow and peristalsis, causing mucosal ischemia. To counteract this, the intestine increases energy metabolism through the AMPK pathway. It also upregulates antioxidant enzymes to protect against oxidative damage (70–72). Short-term corticosterone elevation helps with high-temperature acclimation by redistributing energy. However, prolonged corticosterone activation damages intestinal function. It disrupts the tight junction proteins occludin and ZO-1 in the intestinal epithelium, as well as the mucus layer protein MUC2. This leads to intestinal leakage and microbial imbalance, promoting *Escherichia coli (E. coli*) growth while reducing *Lactobacillus* levels (73, 74).

**Fig 1:**
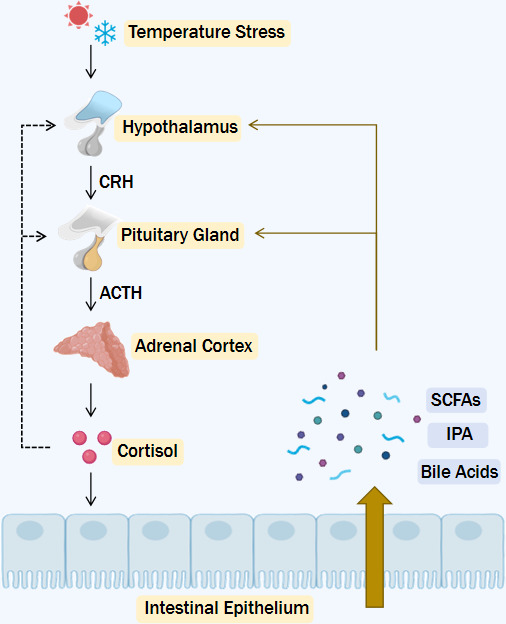
Activation of the HPA axis and feedback regulation by gut microbial metabolites under temperature stress. This schematic illustrates the activation pathway of the HPA axis and the feedback regulation loop mediated by the gut microbiota under temperature stress. Environmental stressors activate the hypothalamic-pituitary-adrenal (HPA) axis, leading to a CRH-ACTH-cortisol cascade that influences the gut microenvironment, thereby altering microbial communities and the production of their metabolites (e.g., SCFAs, IPA, and bile acids). These metabolites act as key signaling molecules that feedback and inhibit the overactivation of the HPA axis via neural or humoral pathways, establishing a complete loop for maintaining organismal homeostasis. In the figure, solid black arrows represent the direct hormone signaling pathway, dashed arrows denote the intrinsic negative feedback within the HPA axis, and solid brown arrows indicate the feedback pathways of microbial metabolites.

Gut microbiota dynamically modulate HPA axis activity through metabolites. Under heat exposure, reduced SCFAs from *Lactobacillus* relieve inhibition on the HPA axis, while indole-3-propionic acid (IPA) enhances epithelial barrier function (75, 76). During cold exposure, *Bifidobacterium*-derived succinate activates intestinal FFAR3 receptors to suppress sympathetic overactivation (77), and tryptophan metabolites (e.g., indolelactic acid) stimulate L cells to secrete glucagon-like peptide-1 (GLP-1), which balances energy compensation via vagal afferent signaling (78, 79). Additionally, microbiota-derived secondary bile acids (e.g., lithocholic acid) activate the nuclear receptor FXR to reduce gut-to-central signaling (Fig. 1) (80). Intestinal fibroblast growth factor 15/19 (FGF15/19) further alleviates HPA axis burden by regulating hepatic metabolism via the portal circulation (81).

This multi-layered regulatory network illustrates that the gut-brain axis, modulated by gut microbial metabolites, serves as a core pathway for sensing and transducing temperature stress signals. By coordinating HPA axis activation, intestinal barrier integrity, and systemic energy metabolism, the microbiota-gut-brain axis enables stable physiological acclimation to temperature fluctuations.

#### Neurotransmitter regulation and neuroinflammation

**Fig 2:**
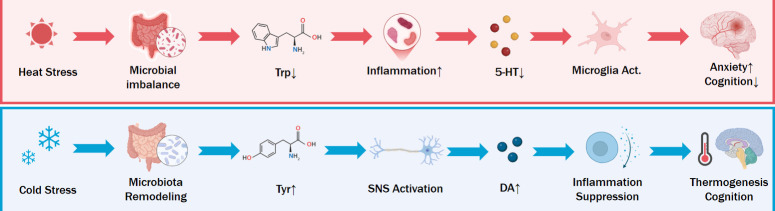
Comparative overview of gut-brain axis-mediated metabolic and neurobehavioral pathways under heat and cold stress.

The gut microbiota dynamically regulates gut-brain axis function through the synthesis of neurotransmitters (e.g., 5-HT, γ-aminobutyric acid [GABA]), and temperature exposure exerts its effects largely by reshaping microbial abundance and metabolic activity that govern neurotransmitter precursor supply (82, 83). Experimental studies demonstrate that chronic heat exposure alters the composition and function of the gut microbiota, which in turn reduces intestinal tryptophan availability and downregulates microbial and host tryptophan hydroxylase (TPH1) expression in intestinal enterochromaffin cells, thereby decreasing colonic and hypothalamic 5-HT bioavailability and increasing anxiety-related behaviors in rodent models (8, 84, 85). Conversely, cold exposure remodels the gut microbiota to enhance tyrosine provision and activates β-adrenergic signaling via microbiota-modulated sympathetic nervous system pathways, boosting tyrosine hydroxylase activity in the adrenal medulla and promoting striatal dopamine synthesis (Fig. 2) (86, 87). These microbiota-driven shifts in neurotransmitter metabolism not only affect neural signaling but also contribute to central inflammatory activation under temperature stress.

Temperature variations further remodel the neuroinflammatory microenvironment through microbiota-dependent systemic signals. Heat exposure disrupts intestinal barrier integrity and alters microbial metabolism, leading to systemic signals that activate central inflammatory pathways in the prefrontal cortex and hippocampus (88). These signals affect central nervous system function by influencing glial activity and neural stability, thereby exacerbating neuronal stress and affecting emotional behaviors (13, 55, 89, 90). Although cold exposure suppresses some inflammatory responses in the forebrain, it induces gut microbial dysregulation and promotes the entry of peripheral factors into the brain through microbiota-related pathways, resulting in reduced hippocampal neurogenesis and altered cognitive function (13).

Gut microbial metabolites simultaneously regulate neurotransmitter balance and neuroinflammation. SCFAs (e.g., propionate, butyrate) maintain gut-brain neurotransmitter homeostasis via GPR41/GPR43, thereby alleviating heat-induced anxiety-related behaviors (91). β-hydroxybutyrate (BHBA) relieves neural stress induced by high temperature (92). Secondary bile acids (e.g., lithocholic acid) regulate glial cell activity via the nuclear receptor FXR, highlighting the critical role of microbial metabolites in coordinating neurotransmission and neuroinflammation during temperature adaptation (93, 94).

Collectively, temperature fluctuations reshape neurotransmitter balance and the neuroinflammatory microenvironment via the gut-brain axis, delineating how environment-microbiota-brain crosstalk coordinates energy metabolism and immune homeostasis during temperature adaptation. This highlights a practical approach: targeting key microbial metabolites such as SCFAs and BHBA offers a holistic strategy to restore neurotransmitter homeostasis and alleviate excessive neuroinflammation, thereby enhancing host neural adaptability to temperature stress.The red pathway illustrates the heat stress response: High temperature induces microbiota imbalance and leaky gut, leading to peripheral tryptophan (Trp) depletion and enhanced systemic inflammation. This results in decreased brain serotonin (5-HT) levels and microglial overactivation, ultimately causing increased anxiety and cognitive impairment. The blue pathway depicts the cold adaptation mechanism: Cold exposure remodels the microbiota, increases tyrosine (Tyr) availability, and activates the sympathetic nervous system (SNS), promoting striatal dopamine (DA) synthesis. Elevated DA suppresses neuroinflammation, thereby maintaining thermogenesis and cognitive stability.

#### Thyroid hormone axis and metabolic regulation

The thyroid hormone axis is finely regulated by intestinal microorganisms and their metabolites, which are key upstream mediators of temperature stress sensing. These convert ambient temperature cues into biochemical signals, cooperating with the HPA axis via gut-brain crosstalk to regulate thyroid metabolism and energy allocation, thereby maintaining metabolic homeostasis under temperature stress. Temperature stress reshapes the gut microbiota and their metabolites (lipopolysaccharides, SCFAs, and indole metabolites), whose signals target peripheral tissues to bidirectionally modulate D2 activity, thereby controlling T4 conversion to active T3 and reshaping thyroid hormone profiles (95).

Under cold stress, cold-acclimated microbiota and their metabolites (e.g., specific SCFAs) promote D2-driven T4 activation, increasing active T3 levels to upregulate UCP1 expression in brown adipose tissue and enhance non-shivering thermogenesis, thereby protecting against cold injury (21, 96). In contrast, heat stress triggers gut dysbiosis and barrier disruption, leading to massive LPS leakage. Circulating LPS, combined with HPA axis-induced high corticosterone, suppresses peripheral D2 activity, restricts T3 synthesis, and reduces basal metabolism to cut invalid energy consumption under high temperature (97).

Microbial metabolites further refine thyroid metabolism: microbially fermented SCFAs promote D2 expression via receptors such as FFAR3 (104) or regulate hepatic FGF21 secretion via nuclear receptor FXR to optimize fatty acid oxidation (98, 99). Meanwhile, *Bacteroides*-derived IPA activates the AhR pathway, stabilizing thyroid hormone rhythmicity and synergizing with AhR-mediated intestinal barrier repair (100). Additionally, temperature stress-induced microbial shifts may regulate intestinal T3AM synthesis, maintaining barrier integrity and fine-tuning aberrant D2 activity (101). In conclusion, gut microbiota and their derivatives form an essential upstream regulatory network connecting the thyroid axis, HPA axis, and neurotransmitter systems. During temperature acclimation, the thyroid axis, as a downstream effector of microbial signals, cooperates with the HPA axis to regulate energy redistribution and thermogenic adaptation, clarifying the core bridging role of gut microbiota and providing theoretical support for microbiota-targeted strategies to alleviate metabolic disorders in stressed animals.

### Regulation of thermogenesis and energy homeostasis

Cold exposure induces significant changes in gut microbiota composition and metabolite production, which play crucial roles in regulating host energy metabolism and thermogenesis in endotherms. For example, cold exposure increases the abundance of specific microbial taxa, such as members of the *Clostridiales* order, which enhances the production of SCFAs such as butyrate and acetate. These SCFAs activate GPCRs and upregulate the expression of thermogenic genes, such as uncoupling protein 1 (UCP1), in BAT (102, 103). UCP1, located in the mitochondrial inner membrane of brown adipocytes, converts the proton gradient in the electron transport chain into heat, thereby facilitating non-shivering thermogenesis (NST) (104–106). Ultimately, this microbial mechanism enhances heat production and helps endotherms maintain body temperature in cold environments. Notably, cold exposure promotes the “browning” of WAT, a process in which subcutaneous WAT transforms into beige-like adipocytes. This transformation is heavily influenced by microbial metabolites, as demonstrated by a study showing that specific gut microbes (such as *Lactobacillus acidophilus* ZJ617) can promote the browning of WAT by producing microbial metabolites (such as spermidine). This process improves metabolic health by activating thermogenic genes (such as UCP1) and β-adrenergic receptors (ADRB3) (107). Studies have shown that mice lacking ACLY in adipose tissue (AclyFAT KO mice) fail to form beige or brown adipocytes under cold exposure, accompanied by reduced expression of UCP1 and other thermogenic genes (108). These findings further highlight the essential role of microbial-metabolic pathways in cold-induced adipose tissue remodeling and systemic thermoregulation.

Heat exposure also alters gut microbiota composition and metabolite production, thereby affecting host energy metabolism. Heat exposure reduces microbial diversity and increases the abundance of *Firmicutes*, which are associated with higher butyrate production. Butyrate, a key microbial metabolite, enhances thermogenesis by activating peroxisome proliferator-activated receptor gamma coactivator 1-alpha (PGC-1α) in brown adipose tissue through the GPR43 signaling pathway (109, 110). Similarly, another SCFA, acetate, promotes mitochondrial biogenesis and the “browning” of WAT by upregulating the expression of genes such as Nrf-1, TFAM, PGC-1α, and electron transport chain-related genes (e.g., Ndufa1, COX IV, ATP5o) (99). Gut microbiota and their metabolites therefore coordinate host energy metabolism and thermogenesis across temperature fluctuations. Through modulating thermogenic gene expression and driving WAT browning, microbial signals translate ambient temperature cues into physiological adjustments of energy expenditure and heat production. These mechanisms underscore the importance of gut microbial regulation in host thermal adaptation and suggest that targeted microbiota manipulation can be applied to stabilize metabolic homeostasis and improve temperature stress tolerance.

### Microbial regulation of the immune system

Temperature stress significantly impacts the immune system by disrupting the homeostasis between gut microbiota and the host, making the intestine more susceptible to pathogenic infections (111). Both heat and cold exposure trigger distinct immune responses, leading to changes in gut barrier function, inflammatory signaling, and oxidative stress. These alterations are closely linked to microbial activity and metabolite production, highlighting the critical role of gut microbiota in modulating host immunity under temperature change.

#### Cold exposure and immune modulation

Cold exposure induces time-dependent immune remodeling largely driven by gut microbial community shifts. Acute cold stress rapidly disturbs intestinal microbial structure and metabolic output, and these microbe-derived signals further trigger NF-κB activation and Th1/Th2 imbalance, marked by elevated pro-inflammatory mediators (iNOS, COX-2, TNF-α) and altered cytokine profiles (decreased IFN-γ with increased IL-4), which collectively constitute a microbe-associated adaptive mechanism to low-temperature challenge (112–115). Short-term cold exposure remodels beneficial microbial consortia to constrain excessive inflammatory cascades, thereby exerting intestinal protective effects (116). In contrast, chronic cold exposure triggers obvious gut dysbiosis, which collaboratively exacerbates sustained intestinal damage through coordinated mechanisms involving heat shock protein (HSP70) dysregulation, apoptosis pathway activation (caspase-3, Bax/Bcl-2), persistent cytokine release (IL-1β, IL-6, IL-18), and NLRP3 inflammasome-mediated pyroptosis (117–120). Notably, chronic cold exposure also disturbs microbial biotransformation of bile acids, reducing taurohyodeoxycholic acid (THDCA) levels and further promoting NLRP3 inflammasome activation and immune imbalance (6). The recent identification of SIRT2 as a critical regulator of cold-induced intestinal barrier dysfunction reveals an important host intrinsic pathway that is highly susceptible to microbial metabolic regulation; its genetic deletion restores epithelial integrity through enhanced autophagy (121), providing novel mechanistic insights and potential therapeutic avenues for cold-associated pathologies. Gut microbiota and their metabolites act as central mediators in these processes. SCFAs generated by specific members of *Clostridiales* help maintain intestinal barrier integrity and restrain excessive inflammation by regulating tight junction protein expression and suppressing overactivation of immune signaling pathways (122). Meanwhile, cold-adapted gut microbiota redirect tryptophan metabolism away from inflammatory pathways, further contributing to immune and redox homeostasis. This bidirectional crosstalk between gut microbiota and host immunity underlies the integrated adaptive response to cold exposure (123).

#### Heat exposure and immune dysregulation

Heat exposure disrupts intestinal tight junctions, increasing permeability and resulting in the excessive accumulation of bacteria, small particles, toxic compounds, and gut microbial metabolites within the intestinal lumen (124–126). This disruption is closely linked to gut microbiota dysbiosis: heat stress shifts the balance of intestinal microbiota, reducing the abundance of beneficial bacteria such as *Lactobacillus* and *Bifidobacterium* while increasing the proportion of opportunistic pathogens like *E. coli* and *Salmonella*. The reduction in beneficial bacteria directly lowers SCFA production, weakening the protective effects of these metabolites on intestinal barrier structure and function (127). The leaked microbial components (e.g., lipopolysaccharides from gram-negative bacteria) and altered metabolites trigger an inflammatory response characterized by the activation of Toll-like receptors (TLRs) and the recruitment of immune cells, particularly macrophages (128, 129).

Transcriptomic analysis of intestinal epithelial cells has revealed significant alterations in pathways associated with phagosome formation, iron homeostasis, and oxidative stress. Notably, the expression levels of key genes involved in immune regulation, including SLC40A1 and SLC11A1, have been shown to be specifically modulated by gut microbial metabolites and microbe-derived signaling molecules (130). Heat exposure also impairs the expression of tight junction proteins (e.g., ZO-1, claudin-1, and occludin) partly through microbial metabolite dysregulation—for instance, reduced levels of short-chain fatty acids (SCFAs) produced by commensal bacteria weaken the protective effects of these metabolites on epithelial barrier integrity (117, 119).

Moreover, heat exposure induces mitochondrial dysfunction, endoplasmic reticulum stress, and NADPH oxidase activation in both host cells and gut microbes, leading to the accumulation of reactive oxygen species (ROS) and oxidative stress. ROS damage lipids, proteins, and DNA, not only in host tissues but also within the microbial community, further exacerbating dysbiosis. This in turn activates the NF-κB and MAPK signaling pathways and amplifies the inflammatory response. Thus, a vicious cycle is formed: heat-induced dysbiosis disrupts the intestinal barrier, promoting microbial translocation and inflammation, which in turn aggravates dysbiosis and systemic inflammation. Throughout this cascade, gut microbial metabolites serve as critical signaling messengers that bridge thermal stress, barrier damage, and immune activation, highlighting their central role in mediating heat-induced immune dysregulation (118, 119, 131, 132).

## TARGETED MICROBIAL INTERVENTIONS TO MITIGATE THE INTESTINAL IMPACTS OF TEMPERATURE FLUCTUATIONS

Temperature change significantly disrupts gut microbiota composition and function, leading to intestinal barrier damage, immune dysregulation, and systemic metabolic disturbances. Given that gut microbiota act as a key mediator in the crosstalk between environmental temperature signals and host physiological responses, targeting the microbiota to develop interventions has become a rational strategy. This is because modifying the structure and function of gut microbiota can effectively reverse the adverse effects caused by temperature-induced microbial dysbiosis, thereby restoring intestinal homeostasis and improving host adaptability.

**Fig 3:**
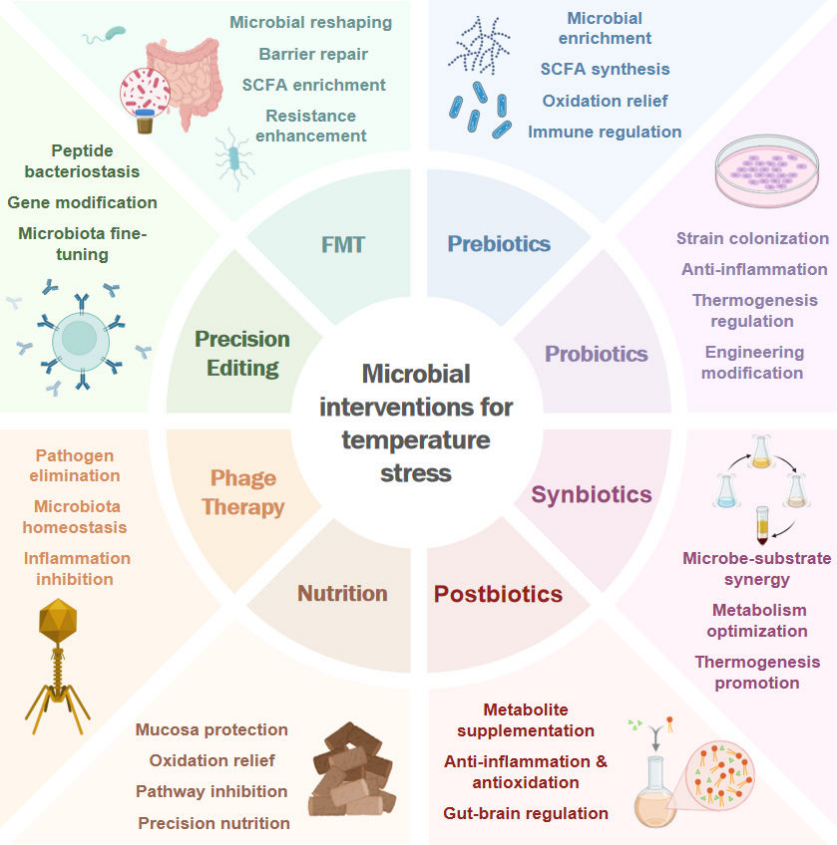
Schematic overview of microbial interventions for mitigating temperature stress in the gut. The diagram summarizes eight major strategies, including FMT, prebiotics, probiotics, synbiotics, postbiotics, nutritional interventions, phage therapy, and precision editing. Each strategy is paired with its core functional mechanisms, such as microbial reshaping, barrier repair, SCFAs enrichment, anti-inflammatory effects, and gut-brain axis regulation, illustrating how targeted microbial and nutritional approaches can restore intestinal homeostasis and enhance host resilience to thermal challenges.

To address these challenges, targeted microbial interventions have emerged as promising strategies to restore gut microbiota homeostasis and enhance host resilience to temperature change. These approaches mainly include FMT, as well as three widely studied microbial-based interventions with distinct mechanisms. Specifically, probiotics are live microorganisms that confer health benefits to the host when administered in adequate amounts, while prebiotics are non-digestible compounds that selectively stimulate the growth or activity of beneficial gut bacteria. Synbiotics, by contrast, combine probiotics and prebiotics to exert synergistic effects that promote gut health more effectively than either alone (133). These interventions aim to modulate microbial communities, strengthen the intestinal barrier, and regulate immune and metabolic responses, thereby improving growth performance and overall animal health under fluctuating temperature conditions (Fig. 3) (134).

### FMT: restoring microbial balance

FMT holds significant potential in alleviating intestinal damage caused by temperature change, particularly in modulating key microbial populations. For example, research indicated that FMT could improve intestinal barrier function by increasing the abundance of butyrate-producing bacteria, such as *Faecalibacterium prausnitzii*, *Blautia*, and *Ruminococcus* (135). These bacteria enhance the intestinal mucosal barrier through the production of SCFAs, reducing inflammatory responses and thereby mitigating intestinal damage induced by heat or cold exposure (136). Additionally, another study highlighted that FMT with gut microbiota from resilient individuals capable of adapting to temperature fluctuations can enhance the host’s immune response and metabolic adaptability by regulating specific microbial populations, such as *Akkermansia muciniphila* and *Bifidobacterium* (137). These microbes play a crucial role in maintaining intestinal homeostasis and regulating the host’s adaptive capacity to temperature change. For instance, *Akkermansia muciniphila* protects the intestinal barrier by modulating the integrity of the mucus layer, while *Bifidobacterium* alleviates stress-induced inflammation through immune regulation. Compared with conventional FMT, constructing standardized synthetic microbial communities (SMCs) from thermotolerant animals offers a more controllable and reproducible alternative for restoring gut homeostasis under temperature stress (138).

### Probiotics: enhancing microbial resilience

Probiotics, defined as live microorganisms that confer health benefits to the host, have been widely used to mitigate temperature change-induced gut dysbiosis. Specific strains of *Lactobacillus*, *Bifidobacterium*, and *Bacillus* have demonstrated efficacy in improving gut barrier integrity, reducing inflammation, and enhancing immune responses under both cold and heat exposure conditions (139, 140). For example, supplementation with *Lactobacillus rhamnosus* in heat-exposed chickens restored gut microbiota diversity, reduced the abundance of pathogenic bacteria, and improved growth performance (141). Similarly, *Lactobacillus plantarum* supplementation in cold-exposed pigs enhanced SCFA production, promoted thermogenesis, and alleviated intestinal inflammation (142). Beyond conventional probiotics, engineered probiotics designed to secrete butyrate, antioxidants, or anti-inflammatory peptides enable more precise and condition-responsive regulation of intestinal homeostasis during temperature stress (143, 144).

### Prebiotics and synbiotics: modulating microbial metabolism

Prebiotics, such as fructooligosaccharides (FOS), galactooligosaccharides (GOS), and inulin, serve as substrates for beneficial gut bacteria, promoting their growth and metabolic activity (145). Synbiotics, which combine probiotics and prebiotics, offer synergistic effects by simultaneously introducing beneficial microbes and providing substrates for their proliferation. Under temperature change, prebiotics and synbiotics have been shown to enhance SCFA production, strengthen the intestinal barrier, and modulate immune responses (146). For instance, dietary supplementation with GOS in heat-exposed broilers increased the abundance of *Lactobacillus* and *Bifidobacterium*, improved gut barrier function, and reduced oxidative stress (147). Synbiotic formulations containing *Lactobacillus acidophilus* and FOS in cold-exposed mice promoted thermogenesis and improved energy metabolism (148). These findings underscore the potential of prebiotics and synbiotics as targeted strategies to modulate microbial metabolism and enhance host resilience to temperature change.

### Postbiotics: harnessing microbial metabolites

Postbiotics are defined as bioactive compounds derived from microbial metabolism. Supplementation with postbiotics represents a novel strategy to mitigate gut dysbiosis induced by temperature stress. Key postbiotics, such as SCFAs, bile acids, and microbial-derived neurotransmitters, play critical roles in regulating host energy metabolism, immune function, and gut-brain axis signaling (149). For example, butyrate supplementation in heat-exposed animals has been shown to enhance gut barrier integrity, reduce inflammation, and improve thermoregulation (150, 151). Similarly, microbial-derived tryptophan metabolites, such as indole-3-propionic acid, have demonstrated anti-inflammatory and antioxidant effects in cold-exposed models (152). These findings suggest that postbiotics can serve as targeted interventions to modulate host-microbe interactions and enhance acclimation to temperature change.

### Nutritional interventions: supporting microbial and host health

Nutritional strategies represent an important approach to support gut health under conditions of temperature change. For instance, glutamine supplementation in heat-exposed animals improved intestinal morphology and reduced inflammation, while N-acetylcysteine alleviated oxidative stress and restored microbial diversity in cold-exposed models (153–155). The addition of glucose to feed can alleviate cold exposure-induced intestinal damage (156). Dietary glucose improves intestinal morphology (increased villus height and reduced crypt depth), inhibits the TLR4/MyD88 pathway, and reduces the expression of inflammatory factors (IL-6, IFN-γ), thereby protecting intestinal barrier function. When combined with microbiome-targeted strategies, these nutritional approaches form a comprehensive system to alleviate temperature-induced gut dysbiosis and enhance host resilience (156, 157). Precision nutrition tailored to temperature-responsive microbial phenotypes further improves the effectiveness of such interventions (157, 158).

### Emerging targeted strategies: novel microbiome-based interventions

Beyond conventional microbiome interventions, emerging targeted strategies offer new, highly specific approaches to alleviate intestinal injury and metabolic disorders caused by environmental stress. These precision interventions show considerable potential to improve gut health and enhance host tolerance under temperature fluctuations.

As a highly selective microbial regulation tool, phage therapy can specifically eliminate pathogenic and opportunistic pathogenic taxa that tend to overproliferate under temperature stress. Phages specifically target undesirable bacteria such as *E. coli* and *Enterococcus faecalis* without disrupting beneficial commensal microbes. This selective removal helps stabilize gut microbial structure, relieve intestinal inflammation, and reduce barrier leakage (159, 160). Similarly, antimicrobial peptides (AMPs) also act as effective protective molecules against stress-induced intestinal damage. These peptides can suppress the growth of pathogenic bacteria enriched during temperature stress, maintain the structure and function of intestinal tight junction proteins, and regulate mucosal immune responses. By strengthening intestinal barrier integrity and inhibiting excessive inflammation, AMPs help maintain intestinal homeostasis under stress conditions (161, 162). Furthermore, CRISPR-Cas-based microbial editing provides a site-specific strategy to precisely remodel the gut ecosystem. This technique can silence virulence genes in conditional pathogens, degrade harmful metabolites, or enhance the metabolic activity of beneficial microbes, enabling fine-tuned regulation of the gut microbiota to better cope with temperature-related stress (163, 164).

Collectively, these precision strategies target key pathological processes of gut dysbiosis induced by temperature stress rather than causing non-specific shifts in microbial composition. Combined with traditional nutritional and microbiome interventions, they can effectively protect intestinal health and improve host adaptability under extreme temperature environments.

## CONCLUSIONS

Temperature fluctuations affect the composition and functionality of the gut microbiota, thereby influencing the host’s energy metabolism, immune function, and intestinal health. In both cold and high-temperature environments, animals adapt to temperature changes through various physiological mechanisms, with the gut microbiota playing an indispensable role in these processes. The gut-brain axis plays a pivotal role in the body’s response to external environmental stress. Temperature stress modulates the composition and metabolic products of the gut microbiota, thereby regulating the synthesis and release of neurotransmitters, which in turn alters the physiological state and emotional responses of the host. Targeted microbial interventions can significantly improve intestinal health under stress conditions and enhance an animal’s ability to adapt to temperature stress. In summary, understanding the mechanisms by which temperature stress influences gut microbiota is critical for revealing the adaptive strategies of animals facing environmental changes and provides a theoretical foundation for developing microbiota-targeted approaches to improve thermal resilience. Future research should further explore the long-term adaptive interactions between temperature stress, gut microbiota, and host physiology and translate these basic mechanistic insights more effectively into practical applications for livestock and poultry production. In particular, efforts should be directed toward developing climate-adapted probiotic preparations, precision nutritional strategies, and gut health regulation technologies suitable for commercial farming systems. Expanding research from laboratory models to intensively farmed livestock, poultry, and even wild populations will help establish more robust and resilient breeding systems, thereby reducing production losses caused by extreme temperature events, enhancing animal stress resistance and production performance, and supporting the sustainable development of animal agriculture under the challenges posed by global climate change.
